# Early Levothyroxine Treatment for Subclinical Hypothyroidism or Hypothyroxinemia in Pregnancy: The St Carlos Gestational and Thyroid Protocol

**DOI:** 10.3389/fendo.2021.743057

**Published:** 2021-10-19

**Authors:** Isabelle Runkle, María Paz de Miguel, Ana Barabash, Martin Cuesta, Ángel Diaz, Alejandra Duran, Cristina Familiar, Nuria García de la Torre, Miguel Ángel Herraiz, Nuria Izquierdo, Ángel Diaz, Clara Marcuello, Pilar Matia, Verónica Melero, Carmen Montañez, Inmaculada Moraga, Natalia Perez-Ferre, Noelia Perez, Carla Assaf-Balut, Miguel Ángel Rubio, Jorge Gabriel Ruiz-Sanchez, Concepción Sanabria, María José Torrejon, Johanna Valerio, Laura del Valle, Alfonso Calle-Pascual

**Affiliations:** ^1^ Endocrinology and Nutrition Department, Hospital Clínico Universitario San Carlos and Instituto de Investigación Sanitaria del Hospital Clínico San Carlos (IdISSC), Madrid, Spain; ^2^ Medicina II Department, Facultad de Medicina, Universidad Complutense de Madrid, Madrid, Spain; ^3^ Centro de Investigación Biomédica en Red de Diabetes y Enfermedades Metabólicas Asociadas (CIBERDEM), Madrid, Spain; ^4^ Gynecology and Obstetrics Department, Hospital Clínico Universitario San Carlos and Instituto de Investigación Sanitaria del Hospital Clínico San Carlos (IdISSC), Madrid, Spain; ^5^ Clinical Laboratory Department Hospital Clínico Universitario San Carlos and Instituto de Investigación Sanitaria del Hospital Clínico San Carlos (IdISSC), Madrid, Spain

**Keywords:** pregnancy, thyroid hormone, subclinical hypothyroidism, hypothyroxinemia, levothyroxine treatment, maternal outcomes

## Abstract

The optimal maternal levels of thyroid hormones (TH) during the first trimester of gestation have not been established, nor has the ideal moment to initiate levothyroxine treatment (LT) to improve the evolution of gestation and fetal development. Cut-off points for Thyroid-stimulating hormone (TSH) <2.5 µIU/mL and free thyroxine (FT4)>7.5 pg/mL have been recommended. There are no data on whether initiation of LT <9th Gestational Week (GW) can have a favourable impact.

**Objective:**

To define the TSH/FT4 percentiles corresponding with 2.5 µIU/mL and 7.5 pg/mL levels, respectively, at GW8 (Study 1), and evaluate the effects of protocol-based LT before GW9 on gestation evolution, in women with TSH ≥2.5 µIU/mL and/or FT4≤ 7.5 pg/mL (study 2).

**Subjects:**

2768 consecutive pregnant women attending the first gestational visit from 2013-2014 and 3026 from 2015-2016 were eligible for Study I and 2 respectively. A final 2043 (study 1) and 2069 (study 2) women were assessed in these studies.

**Results:**

Study 1: The FT4 level of 7.5 pg/mL corresponds with the 17.9th percentile, a TSH level of 2.5 µIU/mL with the 75.8^th^. Women with TSH ≥2.5 µIU/mL had a history of fetal losses more frequently than those <2.5 (OR 2.33 (95%CI): 1.58-3.12), as did those with FT4 ≤7.5 pg/ml compared to those >7.5 (OR 4.81; 3.25-8.89). Study 2: A total of 1259 women had optimal TSH/FT4 levels (Group 1), 672 (32.4%, Group 2) had suboptimal TSH or T4l, and 138 (6.7%, Group 3) had suboptimal values of both. 393 (58.5%) in Group 2 and 88 (63.8%) in Group 3 started LT before GW9. Mean (SD) GW24 levels were TSH: 1.96 ± 1.22 µIU/mL and FT4: 7.07 ± 1.25 pg/mL. The highest FT4 value was 12.84 pg/mL. The adjusted risk for an adverse event if LT was started early was 0.71 (0.43-0.91) for Group 2 and 0.80 (0.66-0.94) for Group 3.

**Conclusions:**

Early LT in women with suboptimum levels of TSH/FT4 (≥2.5µIU/mL/≤7.5 pg/ml) at or before GW9 is safe and improves gestation progression. These data support the recommendation to adopt these cut-off points for LT initiation, which should be started as early as possible.

## Introduction

The role of thyroid hormones in the development of the central nervous system of the embryo/fetus has long been established (1–3). The maternal thyroid is the exclusive source of thyroid hormone early in pregnancy, since the fetus does not start producing thyroid hormone until weeks 16-20 of gestation (4). Of particular importance is maternal FT4, crossing the embryo/fetus hematoencephalic barrier more readily than FT3. In fact, FT4 is the main source of FT3 in the embryo/fetal central nervous system, following FT4 deiodination (4). To assure adequate FT4 delivery to the embryo/fetus, maternal FT4 secretion increases during pregnancy, with a maximum circulating level reached between weeks 7-11 of gestation (4), in spite of a marked increase in thyroxine-binding globulin (TBG).

When the maternal thyroid is compromised, embryo/fetal development can be affected. Descendants of mothers with hypothyroxinemia during the first trimester of gestation, isolated or associated with inadequate Thyroid-stimulating hormone (TSH) levels, have been found to present a reduced Intelligence quotient (IQ) and delayed neurocognitive development (5–8), together with other alterations (9–11). Hypothyroxinemia and subclinical hypothyroidism are also associated with adverse events during pregnancy, such as early fetal loss, prematurity, fetal developmental alterations (12–14), gestational diabetes mellitus (GDM), preeclampsia and hypertension (13, 15). However, the effect of levothyroxine therapy (LT) on pregnancy outcomes is controversial. Furthermore, levothyroxine therapy could induce overtreatment and overdose, also harmful (6).

The timing of levothyroxine treatment could **be** crucial for improvement of pregnancy outcomes. Fluctuations in thyroid hormone levels (TH) can be seen during the first weeks of gestation (16, 17). Animal models suggest that normalization of thyroid hormone levels (TH) with levothyroxine after the equivalent of the human 10^th-^13^th^ gestational week does not correct neurological alterations induced by hypothyroxinemia (18–20). Yet in humans it takes several weeks of oral LT before a steady state of FT4 can be reached. The extrapolation to humans of findings in animal models would suggest that there is a “window of opportunity” for initiation of LT to induce maximum benefits on fetal development, with LT starting ideally weeks before the end of the first trimester of gestation. In fact, recommendations for pregnant women with gestational thyroid dysfunction have included the start of levothyroxine therapy as early as possible, and before the 9^th^-10^th^ gestational week (GW) (20–23). Yet the GW in which FT4/TSH values should **be** obtained, permitting initiation of LT, is a point of controversy (8, 22–29).

In 2013, when this study was initiated, levels of TSH <2.5 µIU/mL and FT4 >7.5 pg/mL **were** considered appropriate during the first trimester of pregnancy (6), and cut-off points for initiation of LT. However, the use of a specific reference range from the local disease-free population is currently recommended (30).

To obtain a local TH reference range, TSH and FT4 values from all pregnant women who attended prenatal screening consultation in our hospital from 2013-2014 were analyzed. Furthermore, a treatment protocol for subclinical hypothyroidism (SCH) and isolated hypothyroxinemia was applied starting in 2015, based on the 2014 ATA (21) and ETA (22) recommendations, with initiation of LT as soon as elevated TSH and/or FT4 levels were detected. We present the distribution of TSH/FT4 levels found in our population, and the effect of levothyroxine therapy on adverse maternal-fetal events. A comparison of the results on pregnancy progression as a function of the timing of LT initiation was also undertaken.

## Materials and Methods

### Experimental Design

The Hospital Clínico San Carlos covers public-system healthcare for 455,000 inhabitants in the central area of Madrid, Spain. Prenatal screening testing is universal, and centrally performed, at approximately GW10-12.

#### Study 1: The Reference Range

From 2013-2014, 2768 pregnant women with a single, spontaneous pregnancy, and no history of prior disease attending prenatal screening consultation before GW12 were included in the study. Patients with pregestational fertility treatment or known diseases were excluded. Clinical characteristics of the study population are shown in **Table S1**. To establish the reference range, women whose TH had been obtained at GW 8 ± 1 were selected, based on the gestational age obtained at the first ultrasound study. Four hundred forty-one women with TSH/FT4 levels determined after GW9 were excluded. A further 248 women were excluded: women diagnosed with pregestational thyroid disease or on prior levothyroxine treatment, as well as those with overt thyroid dysfunction at GW 8 ± 1 as defined by our laboratory’s reference range for non-pregnant adults (women with TSH >5.3 µIU/mL or FT4 <5.8 pg/mL or both for hypothyroidism, and those with a TSH <0.3 µIU/mL combined with a FT4 >16.4 pg/mL for hyperthyroidism). Following exclusions, a sample of 2043 women was obtained and evaluated. Information on obstetric history, pregestational diseases and drug treatments, and iodized salt use was collected.

#### Study 2: Application of the Protocol of Thyroid Dysfunction Management During Pregnancy

During 2014, an intervention protocol for LT in the presence of first trimester thyroid hormone alterations was applied.

In summary, and based on ATA and ETA 2014 GUIDELINES (21, 22), TSH levels ≥2.5 µIU/mL were considered indicative of subclinical hypothyroidism and FT4 ≤7.5 pg/ml of hypothyroxinemia, and susceptible to treatment. Levothyroxine doses were as follows: when TSH was 2.5-3.4 µIU/mL, the initial dose was 50 mcg/day, and was 100 mcg/day when TSH was 3.5-5 µIU/mL. With TSH levels >5 µIU/mL, LT was initiated with a loading dose of 2-2.4 g/kg/day, approximately 150 g/day, during the first week. If FT4 levels were ≤7.5 pg/mL, the initial LT dose was 50 mcg/day when TSH levels were 0.2-2.5 µIU/mL, or 100 mcg/day with TSH ≥2.5 µIU/mL. If FT4 was < 6 ng/dl, the initial dose was 100 mcg/day regardless of TSH levels. Any value of FT4 > 14 pg/ml at GW24-28 was classified as overdose. The diagnostic and treatment protocol can be seen in “supplementary Appendix 1” in English and in the original version. Optimal timing for initiation of therapy was considered to be as early as possible, at the time of confirmation of gestation and before GW9.

### Women Studied

Between 2015 and 2016, a total of 3026 consecutive women who attended the prenatal screening visit before GW12, with a singleton pregnancy and without prior fertility treatment or known diseases, were initially eligible for the study. Characteristics can be seen in **Supplementary Table S1**. Women diagnosed with pregestational thyroid disease or on prior levothyroxine treatment as well as those with overt thyroid dysfunction at GW 8 ± 1 as defined by our laboratory’s reference range for non-pregnant adults as specified above for Study 1 (n=135), and those whose TH determination was obtained later than GW8 ± 1 (n=822) and therefore not likely to receive early replacement treatment were excluded. A final 2069 women were included for analysis.

During the first prenatal visit, relevant demographic and clinical data were obtained including age, ethnicity, parity, educational level, salaried work, declared pregestational body weight, smoking habit, family and personal history of metabolic disease, history of fetal losses, and consumption of iodized salt. At that visit, an ultrasound study was conducted to confirm gestation, and the age obtained was considered the gestational age.

### Clinical Information and Follow-up During Pregnancy

Fetal losses prior to GW12 and between GW12-18 were analysed. Voluntary pregnancy interruptions were not included. Birth before GW32 was considered immaturity, and before the 37^th^ considered preterm birth. GDM was diagnosed following an oral 75 g glucose overload performed between GW24-28, in accordance with the diagnostic criteria of IADPSG, adopted by WHO. Pre-eclampsia was considered when blood pressure was ≥140/90 mm Hg after GW20 and albuminuria >300 mg/day. Delivery was classified as non-instrumental vaginal, instrumental or Caesarean-section. Newborns were categorized as small or large for Gestational age (<p10 or >p90, respectively) according to local growth tables. They were included in the variable of births with inadequate weight for gestational age, whether large or small for gestational age. A variable composed of suffering at least one adverse event during pregnancy was calculated. It included pre-eclampsia, GDM, fetal loss, immature and/or premature newborn, Cesarean section, and newborn of inadequate weight for Gestational age.

### Thyroid Tests

TSH was measured by a 3^rd^ generation sandwich-chemiluminescence immunoassay with magnetic particles using human TSH mouse monoclonal antibodies in a DXI-800^®^ (Beckman –Coulter). The manufacturer’s stated normal range for non-pregnant adults is 0.38-5.33 µIU/mL. Sensitivity 0.01 µIU/mL, intraassay coefficient of variation (CV) <10% and range 0.01-50.0 µIU/mL Intra-assay CVs are 4.9% for a concentration of 0.69 µIU/mL, 5.8% for 5.47 µIU/mL and 6.2% for 29 µIU/mL, respectively.

FT4 was measured by a competitive-chemiluminescence immunoassay in 2 steps with paramagnetic particles, in a DXI-800^®^ (Beckman –Coulter). The manufacturer’s stated normal range for non-pregnant adults is 5.8–16.4 pg/mL. Sensitivity 2.5 pg/mL, range: 2.5 – 60 pg/mL. Intra-assay CVs are 7.8% for a concentration of 8.5 pg/mL, 5.7% for 22.9 pg/mL and 4.3% for 43 pg/mL, respectively.

Anti-TPO antibodies were determined in women with abnormal TSH and/or FT4 levels, using a sandwich-type microplate ELISA test to measure IgG antibodies against thyroid peroxidase, Anti-TPO- ORGENTEC Diagnostika GmbH. Sensitivity 5 IU/ml, range: 5-3000 IU/ml L. Intra-assay CV are 3.1% for a concentration of 324 IU/ml, 3.5% for 761 IU/ml and 9.7% for 2173 IU/ml, respectively.

Evaluation of all analytical methods is performed through the SEQC Quality Assurance Program, external monthly periodicity control.

### Ethics Statement

The study was performed in compliance with the Declaration of Helsinki, approved by the Ethics Committee of Hospital Clínico San Carlos (13/296-E and 14/298-E_BS) and the use of thyroid function data (18/484-E_BS). All women signed a letter of informed consent.

### Statistical Study

Continuous variables are shown as mean and SD and/or median and interquartile range, and categorical variables as number and percentage. The Saphiro–Wilk test was used to verify the normal distribution of the data. Means were compared by Student´s T test, and medians by the Mann-Whitney U test, with categorical variables compared by the chi-squared test. The population distribution of TH obtained at GW8 ± 1 is expressed in percentiles and frequency distribution.

Logistic regression analysis was performed to assess the adverse effects of having a TSH and/or FT4 outside the levels considered optimal as compared to having both in the range considered normal. The magnitude of association was evaluated using the odds ratio (OR) and 95% confidence interval (95%CI). For early treatment assessment, the OR of adverse events was calculated comparing LT initiation both before or after the 9^th^ gestational week with women whose values of both hormones were normal (reference group). Reduction in OR was also assessed comparing start of LT before the GW9 to LT initiated later (reference group) in pregnancy. Multivariate analysis was adjusted for age, parity and smoking status.

All p values are 2-tailed at <0.05. Analyses were performed using SPSS, version 21 (SPSS, Chicago, Illinois).

## Results

**Study 1.** **Table 1** shows the TSH and FT4 distribution percentiles of the 2043 women studied, with distribution frequencies in **Figure S1**.

**Table 1:** Thyroid hormone levels by percentiles in the population studied in gestation week 8 ± 1.

Percentile	1	2.5	5	25	50	75	95	97.5	99
**TSH µIU/mL**	0.06	0.15	0.34	1.09	1.71	2.47	3.85	4.29	4.67
**FT4 pg/mL**	5.85	6.31	6.69	7.80	8.56	9.47	10.97	11.67	12.89

Two hundred forty-six out of 573 (42.9%) women with TSH levels ≥2.5 µIU/mL had a history of prior fetal losses as compared with 299 of 1496 (20%) women with TSH <2.5 µIU/mL. Adjusted OR for fetal losses was 2.33 (1.58-3.12). Two hundred eighty-six of 404 (70.8%) women with FT4 levels ≤7.5 pg/mL had a prior history of fetal losses, with the latter observed in only 395/1665 (23.7%) with FT4 levels >7.5 pg/mL The adjusted for age, parity, and smoking habit OR for fetal losses was 4.81 (3.25-8.89) with FT4 ≤7.5 pg/mL (**Table 2**).

**Table 2:** Odds ratio (95% confidence interval) for a history of prior fetal losses adjusted for age, parity, and smoking habit in women from **Study 1**.

Women with	History of prior fetal losses		
	N events/total (%)	OR	95% CI
**TSH <2.5 µIU/mL**	299/1496 (20.0)	Reference	
**TSH ≥2.5 µIU/mL**	246/573 (42.9)	2.33	1.58-3.12
**FT4 >7.5 pg/mL**	395/1665 (23.7)	Reference	
**FT4 ≤7.5 pg/mL**	286/404 (70.8)	4.81	3.25-8.89

**Study 2.** Of the 2069 women assessed in the study, 1259 (Group 1) had TSH levels <2.5 µIU/mL and FT4 > 7.5 pg/mL, while 672 (32.4%; Group 2) had one inadequate value, and 138 (6.7%; Group 3) presented both values outside of optimal levels. Their characteristics are shown in (**Table 3**).

**Table 3:** Characteristics of the women studied in **Study 2**.

	Group 1	Group 2	Group 3	P value
	TSH<2.5µIU/mL and FT4 >7.5 pg/mL	TSH≥2.5µIU/mL or FT4 ≤ 7.5 pg/mL	TSH≥2.5µIU/mL and FT4 ≤ 7.5 pg/mL	
**Total N 2069**	1259 (60.9%)	672 (32.4%)	138 (6.7%)	
**Age (yr)**	33.05 (5.12)	32.21 (5.20)	32.03 (5.29)	0.001
**HPFL**	421 (33.4%)	229 (34.0%)	56 (40.6%)	0.032
**Caucasian Ethnicity**	879 (69.8%)	415 (61.8%)	68 (50.0)	0.001
**Primiparous**	533 (42.5%)	304 (45.4%)	48 (34.8)	0.043
**University degree**	845 (67.2%)	321 (62.6%)	75 (54.3%)	0.005
**Salaried work**	1011 (80.4%)	513 (76.3%)	104 (75.4%)	0.412
**No prior history of any metabolic disease**	1027 (81.6%)	554 (82.4%)	96 (69.6%)	0.020
**No family history of any metabolic disease**	325 (25.8%)	176 (26.2%)	45 (32.6%)	0.017
**Smoker until**	175 (13.9%)	66 (9.8%)	18 (13.0%)	0.028
**/during pregnancy**	111 (8.8%)	52 (7.7%)	10 (7.2%)	
**Body Weight (Kg)**	61.68 (11.11)	62.59 (12.04)	62.00 (9.79)	0.246
**BMI (Kg. m^-2^)**	23.21 (3.97)	23.84 (4.34)	23.96 (3.66)	0.002
**TSH (μIU/mL)**	1.35 (0.64)	2.95 (1.56)	4.16 (2.71)	0.000
**Mean (SD)** **Median (Q_1-3_)**	1.36 (0.89-1.89)	2.77 (1.94-3.55)	3.36 (2.93-4.20)	0.000
**FT4 (pg/mL)**				
**Mean (SD)**	9.16 (1.30)	8.07 (1.37)	6.68 (1.04)	0.000
**Median (Q_1-3_)**	8.95 (8.23-9.77)	8.02 (7.24-8.87)	6.92 (6.50-7.24)	0.000
**A-TPO ab >50 IU/mL**	N.A.	116 (17.3%)	19 (13.8%)	0.741
**Use of Iodized salt**	522 (41.5%)	278 (41.4%)	69 (50.0%)	0.685

Results expressed as number (%) or mean (SD); HPFL, History of prior fetal loss; BMI, Body mass index; A-TPO ab, Antithyroid peroxidase antibodies.

Three hundred ninety-three of the 672 women (58.5%) of Group 2, and 88 women (63.8%) of the 138 of Group 3 began LT before the GW9, while 183 (27.2%) from group 2 and 31(22.5%) from group 3 began LT between GW10-24. Ninety-six (13.8%) women from Group 2 and 19 (13.8%) from group 3 respectively, either did not receive LT or started it later. The occurrence of events during gestation, as a function of LT initiation prior to or later than GW9, can be seen in **Table 4**.

**Table 4:** Evolution of gestation by groups, and in relation to the beginning of the levothyroxine treatment (LT) before GW9 or later/did not receive it.

	GR0UP 1	GROUP 2			GROUP 3			P value
		ALL	LT		ALL	LT		
			AFTER GW9	BEFORE GW9		AFTER GW9	BEFORE GW9	
**N 2069**	1259	672	279	393 (58.5)	138	50	88 (63.8)	
**PHFL**	419 (33.3)	229 (34.1)	111 (39.8)	118 (30.1)	56 (40.6%)	25 (50.0)	31 (35.2)	0.056
**OR**	REFERENCE	1.03	1.10	0.98	1.36	1.56	1.24	
**95% CI**		0.84-1.25	0.85-1.42	0.76-1.25	0.95-1.95	0.91-2.66	0.78-1.97	
**Miscarriage <12 GW**	20 (1.6)	10 (1.5)	7 (2.5)	3 (0.8)	4 (2.9)	3 (6.0)	1 (1.1)	0.480
**OR**	REFERENCE	0.94	1.22	0.69	1.85	3.55	0.76	
**95% CI**		0.44-2.02	0.85-3.07	0.24-2.05	0.62-5.51	1.02-12.35	0.10-5.75	
**Miscarriage <18 GW**	21 (1.7)	12 (1.8)	9 (3.3)	3 (0.8)	4 (3.0)	3 (6.4)	1 (1.1)	0.604
**OR**	Reference	1.00	1.16	0.87	1.71	3.06	0.74	
**95% CI**		0.49-2.05	0.84-1.27	0.35-2.19	0.57-5.11	0.87-10.81	0.10-5.60	
**Immature <32 GW**	4 (0.3)	8 (1.2)	6 (2.3)	2 (0.7)	1 (0.8)	1 (2.3)	0	0.094
**OR**	Reference	3.54	4.10	3.11	2.21	3.96		
**95% CI**		1.06-11.83	1.02-16.58	0.77-12.54	0.24-20.03	0.43-36.20		
**Prematurity <37 GW**	59 (4.9)	44 (6.5)	26 (10.1)	18 (4.7)	6 (4.7)	3 (6.9)	3 (3.5)	0.148
**OR**	Reference	1.43	1.69	0.87	0.98	1.01	0.78	
**95% CI**		0.96-2.13	1.04-2.77	0.59-1.28	0.91-1.05	0.94-1.04	0.24-2.56	
**VAGINAL N-I**	813 (60.8)	400 (61.5)	164 (62.4)	236 (61.0)	70 (56.0)	31 (60.8)	39 (52.7)	0.504
**OR**	Reference	1.07	1.09	1.01	0.85	1.03	0.74	
**95% CI**		0.87-1.31	0.73-1.13	0.78-1.31	0.58-1.23	0.58-1.84	0.46-1.59	
**Caesarean Section**	227 (18.0)	119 (17.7)	57 (20.4)	62 (15.8)	33 (23.9)	12 (24.0)	21 (23.8)	0.328
**OR**	Reference	0.92	1.05	0.90	1.29	1.11	1.42	
**95% CI**		0.72-1.18	0.81-1.36	0.85-1.39	0.84-1.97	0.57-2.15	0.84-2.41	
**GDM**	235 (18.6)	111(18.8)	54 (20.0)	56 (17.5)	30 (23.3)	10 (23.3)	20 (23.3)	0.391
**OR**	Reference	1.00	1.09	0.93	1.35	1.07	1.06	
**95% CI**		0.77-1.30	0.95-1.53	0.67-1.28	0.87-2.09	0.53-2.16	0.92-2.66	
**Preeclampsia**	12 (1.0)	8 (1.5)	5 (2.0)	3 (1.1)	3 (1.8)	2 (4.2)	0	0.567
**OR**	Reference	1.56	2.06	1.11	1.84	4.48		
**95% CI**		0.60-4.07	0.83-6.20	0.30-4.13	0.39-8.62	0.94-21.32		
**SGA/LGA**	206 (16.9)	131 (23.5)	65 (24.8)	66 (22.4)	27 (20.8)	12 (27.3)	15 (17.4)	0.484
**OR**	Reference	1.16	1.25	1.09	1.09	1.20	1.01	
**95% CI**		0.91-1.49	0.98-1.72	0.80-1.49	0.69-1.71	0.61-2.33	0.56-1.83	
**Composite AO**	296 (23.5)	183 (27.3)	82 (28.8)	101 (25.9)	37 (27.2)	17 (34.0)	20 (22.7)	0.256
**OR**	Reference	1.22	1.32	1.14	1.32	1.77	0.98	
**95% CI**		0.96-1.56	0.99-1.80	0.84-1.55	0.67-1.54	1.05-2.99	0.54-1.77	

Results expressed as number (%); LT, levothyroxine treatment; GW, gestational week; HPFL, History of prior fetal loss; N-I, no instrumental; GDM, Gestational Diabetes Mellitus SGA, Small-for-gestational-age; LGA, large-for-gestational-age; Composite AO, Composite Adverse Outcomes: pre-eclampsia, GDM, fetal loss, immature and/or premature newborn, C-section, and newborn of inadequate weight for Gestational age (SGA and LGA); OR (95%CI), Odds Ratio (95% confidence interval) adjusted for age, parity, and smoking habit.

**Figure 1:**
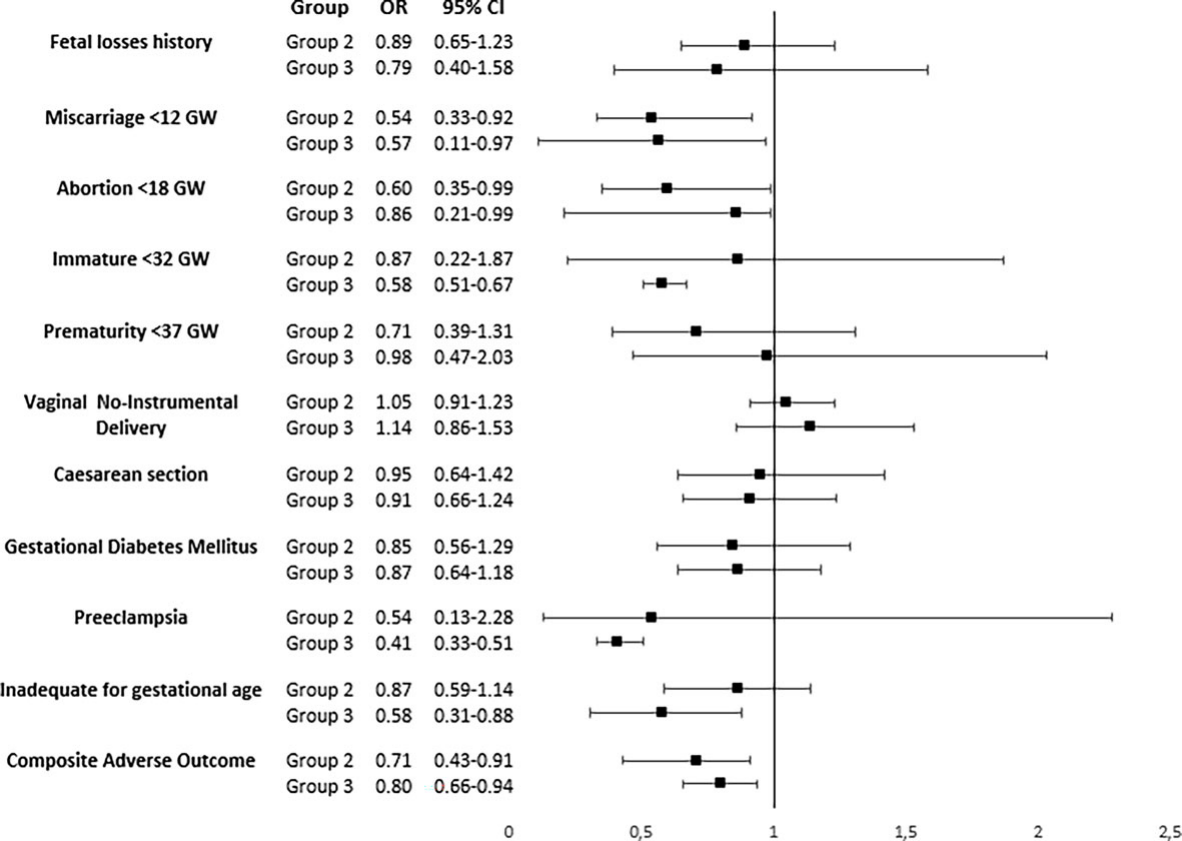
Odds ratio (95% confidence interval) adjusted for age, parity, and smoking habit to receive levothyroxine treatment before week 9th of gestation compared to receiving it later or not to receive it as the reference group, by groups. Composite Adverse Outcomes: pre-eclampsia, GDM, fetal loss, immature and/or premature newborn, C-section, and newborn of inadequate weight for Gestational age.

The reduction in the risk for having at least one adverse event when starting LT before GW9 as compared to a later start can be seen in **Figure 1**.

The adjusted OR for age, parity, and smoking habit in women for suffering at least one event in the composite variable was 0.71 (0.43-0.91) for women in Group 2 and 0.80 (0.66-0.94) for women in group 3 respectively when receiving LT prior to GW9. Evolution of gestation by groups (group 2 and 3) comparing women starting levothyroxine treatment (LT) between 10-24 GW with those not receiving LT or starting after GW 24 (no LT), with Group 1 as reference, is displayed in **Table S2**.

Data for women with TSH ≥2.5 vs. <2.5 µIU/mL can be seen in **Table S3** and for women with FT4 ≤7.5 vs. >7.5 pg/mL in **Table S4**. LT prior to GW9 reduced rates of fetal loss and immature births in women with TSH ≥2.5 µIU/mL, while reducing the rate of preeclampsia and inadequate weight for gestational age newborns in women with FT4 ≤ 7.5 pg/mL. In both groups, early LT significantly reduced the composite variable.

At GW24, average TH levels of treated women were 1.96 ± 1.22 µIU/mL for TSH and 7.07 ± 1.25 pg/mL for FT4. Only 9 women had TSH values <0.10 µIU/mL. None had FT4 levels >14 pg/mL, with the highest value at 12.84 pg/mL.

## Discussion

Optimum neurocognitive development of offspring as well as maternal/fetal wellbeing during pregnancy are important goals when considering initiation of levothyroxine therapy in pregnancy. The current study has found that women with a TSH level ≥2.5 µIU/mL or FT4 ≤ 7.5 pg/mL have a 2 and 4-fold higher risk, respectively, for prior fetal losses as compared with other women. Furthermore, levothyroxine therapy introduced early on in pregnancy, with an intervention cut-off point of 2.5 µIU/mL for TSH and 7.5 pg/mL for FT4, reduced adverse gestational events. In fact, initiation of LT before GW9 reduced rates of fetal loss prior to week 12 by over 40%, with a 14-40% reduction in fetal loss before week 18. Early LT also reduced the risk for premature delivery before GW32 by 14-29%. The rates of preeclampsia and newborns with inadequate weight for their gestational age were also decreased. Early treatment induced a 29% reduction in adverse events during pregnancy in women with one suboptimal value of TH, and 20% in those with suboptimal levels of both TSH and FT4. Benefits in rates of fetal loss and immature births on the one hand, and of preeclampsia and inadequate weight for gestational age on the other were also seen when analyzing women with high TSH levels and low FT4 levels separately, respectively. Yet no cases of overtreatment/overdose were observed. Thus, LT is safe, and can improve maternal/fetal health, with the aforementioned cut-off points for TSH and/or FT4 used as indications for therapy, when initiated before the 9^th^ gestational week.

A previous study by Negro et al. (31) found that women with thyroid dysfunction but no risk factors for thyroid disease who received LT had a statistically significant decrease in overall adverse outcomes. These results are in strong support of the present study.

To start LT before the week 9, TH must be measured early in gestation. A specific timing of TH measurement is preferable for uniform assessment of maternal thyroid function. We selected TH measured in week 8, corresponding with the lowest gestational TSH levels (16).

Although LT initiation before GW9 reduced adverse gestational events, there was no benefit to LT commenced later in gestation. An early start could help mimic the physiological FT4 rise observed between the GW7-11 in iodine-sufficient women (4). In fact, the physiology of embryo/fetal development and brain development chronology (2, 15) indicate that maximum benefits of levothyroxine therapy would be seen when started prior to GW9.

The timing of LT initiation could explain discrepancies between our results and those of prior studies (24, 26, 32–34). Although 2 metaanalyses found that LT had favorable effects on gestational endpoints in women with gestational thyroid hypofunction, a third did not (35–37). However, LT was started later than in the current study in the majority of cases, even up to GW20. To our knowledge, no prior study specifically analyzes pregnancy endpoints in women starting therapy before GW9.

Benefits for maternal/fetal health were found when using the 2014-recommended cut-off points of TSH ≥2.5 µIU/mL and/or FT4 ≤ 7.5 pg/mL as indications for LT, as long as the latter was initiated early. Other studies have also found an association between 1^st^ trimester TSH levels > 2.5 and increased fetal losses (9–11). ATA 2017 guidelines recommend the use of a local reference range to determine normal TH (30). Yet the distribution of TSH/FT4 levels found in our healthy population indicates that the 97.5th percentile of TSH is at 4.29 µIU/mL and the 2.5th percentile of FT4 is at 6.31pg/mL in GW8. Additionally, the guidelines recommend that isolated hypothyroxinemia not be routinely treated. Furthermore, ATA 2017 Guidelines only strongly recommend LT in women presenting 1st trimester TSH levels > 10.0 when anti-TPO antibodies are negative (30). Nor is isolated hypothyroxinemia considered to be an indication for LT. Our results suggest that the application of these recommendations to determine cut-off points for therapy could exclude many women whose pregnancies could benefit from LT when started before GW9.

Maternal thyroid dysfunction as defined by a TSH ≥ 2.5 µIU/mL or a FT4 ≤ 7.5 pg/mL was frequent, highlighting the importance of universal screening for thyroid dysfunction in pregnancy. Fully 32.5% of women exhibited an alteration in either TSH or FT4, with 6.7% exhibiting both. Data obtained in this study show that approximately 30% of pregnant women in Spain would be starting on LT if our results were extrapolated to the rest of Spain. One possible explanation or contributing factor for this high rate could be iodine deficiency. In a population study (38) that we carried out in Spain, urinary iodine in women of childbearing age between 18-49 years ranged from 127-139 µg/L according to decades of age, indicating that Spain is not an iodine-deficient country. However, iodine consumption is not uniform throughout Spain, and there are areas with iodine deficit where iodized salt is only used by less than 50% of the population, figures similar to those found in the current study. In a preliminary analysis (data no shown), we have found that the women included in this study had an average pregestational iodine consumption of 120 µg/day, clearly insufficient during pregnancy. Gestational iodine consumption would only increase once pregnancy is known and supplementary iodine initiated. This pregestational, and early-gestational low iodine intake could be inducing or contributing to the high rate of SCH and/or hypothyroxinemia observed, and the need to start LT as early as possible.

Yet, although the protocol called for initiation of levothyroxine therapy as soon as TH alterations were detected, only 80% of women started treatment before GW24. Furthermore, only 58% and 63% of women with suboptimal TSH and FT4 respectively initiated therapy prior to GW9, presumably due to a delay in the date of the 1^st^ prenatal visit. Iodized salt consumption was low. Our results highlight the need for universal TH screening in women early in pregnancy, with the participation of primary care professionals, together with public health programs designed to improve iodine status.

This study has several limitations. Iodine deficiency was not analyzed, as women were evaluated at gestation week 9-12, when most were taking iodine supplements. Per-protocol assessments can have uncontrolled confusion factors. Women receiving early replacement therapy are seen earlier on in pregnancy, suggesting a better pregnancy follow-up. However, our results reflect real-life clinical practice. Furthermore, results have been adjusted by age, parity and smoking habit, the most influential factors in gestation evolution. The screening and treatment protocol does not include universal measurement of antithyroid antibodies, impeding assessment of a direct effect of thyroid autoimmunity on results. Although the effects of LT on women with TSH ≥ 2.5 µIU/mL, as well as in women with FT4 level ≤ 7.5 pg/mL were independently analyzed, overlap between the two groups occurred.

A main strength of the study is the description of population-specific ranges of thyroid hormones obtained in 8th week of gestation. Another main strength is having carried out an intervention study with levothyroxine in early stages of gestation, prior to the 9^th^ week, which we were able to do since 8^th^-week- thyroid hormone levels were available. Conclusions: Levothyroxine therapy, initiated before GW9, reduces adverse events in pregnancy in women with TSH ≥2.5 µIU/mL and/or FT4 ≤ 7.5 pg/ml. We believe these TH cut-off points should be maintained as indications for LT, TH determined at week 8, and LT initiated before GW9 to optimize maternal/fetal health. Given the high frequency of thyroid hypofunction detected, to assure early detection and therapy of thyroid alterations, we feel that universal screening should take place at approximately week 8 of pregnancy, with participation of primary care professionals. Further studies on the consequences of early therapy should be performed to confirm our findings.

## Data Availability Statement

The raw data supporting the conclusions of this article will be made available by the authors, without undue reservation.

## Ethics Statement

The studies involving human participants were reviewed and approved by the Ethics Committee of Hospital Clínico San Carlos (13/296-E and 14/298-E_BS) and the use of thyroid function data (18/484-E_BS). All women signed a letter of informed consent.

## Author Contributions

Study concept and design, acquisition of data, analysis and interpretation of data, drafting of the manuscript, critical revision of the manuscript for important intellectual content, material support and study supervision: All authors. IR and MPdeM contributed equally to this work. All authors have seen and agree with the content of the last version of manuscript.

## Funding

This research was funded by grants from Fundación para Estudios Endocrinometabolicos, IdISSC Hospital Clínico San Carlos, Universidad Complutense of Madrid, Medicine Department; the Instituto de Salud Carlos III/MICINN of Spain under grant number PI17/01442, and European Regional Development Fund (FEDER) ‘‘A way to build Europe’’ and Sociedad de Endocrinología, Nutrición y Diabetes de la Comunidad de Madrid (SENDIMAD) under grant number IPI/2018/NR5. The design and conduct of the study; collection, management, analysis, and interpretation of the data; preparation, review, and approval of the manuscript; and decision to submit the manuscript for publication are the responsibilities of the authors alone and independent of the funders.

## Conflict of Interest

The authors declare that the research was conducted in the absence of any commercial or financial relationships that could be construed as a potential conflict of interest.

## Publisher’s Note

All claims expressed in this article are solely those of the authors and do not necessarily represent those of their affiliated organizations, or those of the publisher, the editors and the reviewers. Any product that may be evaluated in this article, or claim that may be made by its manufacturer, is not guaranteed or endorsed by the publisher.

## Supplementary Material

Supplementary Figure 1 Distribution of TSH µIU/mL **(A)** and FT4 pg/mL **(B)** levels by frequency, in the studied population at the 8^th^ gestational week (GW).
