# Supplementary material for: Early Levothyroxine Treatment for Subclinical Hypothyroidism or Hypothyroxinemia in Pregnancy: The St Carlos Gestational and Thyroid Protocol
Source: Front Endocrinol (Lausanne). 2021 Oct 19;12:743057. doi: 10.3389/fendo.2021.743057 (PMC8560890; doi:10.3389/fendo.2021.743057)
Supplement: Supplementary Figure 1 — Distribution of TSH µIU/mL (A) and FT4 pg/mL (B) levels by frequency, in the studied population at the 8th gestational week (GW). [file DataSheet_1.pdf]

Figure S1. Distribution of TSH µIU/mL (a) and FT4 pg/mL (b) levels by frequency, in the studied population at the 8th gestational week (GW)

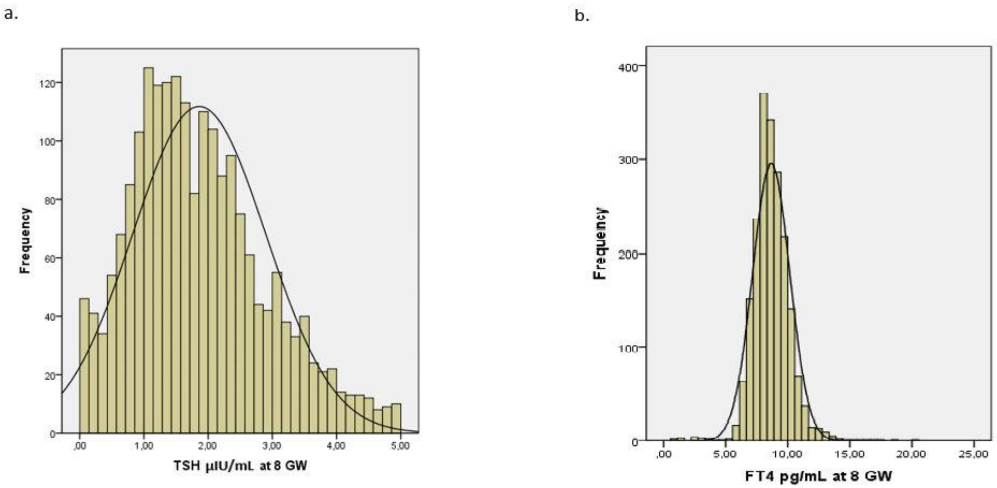

Table S1. Characteristics of women eligible for Study 1 and 2

	Before Protocol 2013-14	After Start of Protocol 2015-16	**P value**
**N**	2768	3026	
**Age (yr) Median (Q_1-Q_3)**<br>**Mean ±SD**	32 (28-36)<br>31.6 ± 5.7	33 (29-36)<br>32.6 ± 5.3	0.001<br>0.001
**History of prior fetal loss**	681 (24.5%)	952 (29.7%)	0.001
**Caucasian Ethnicity**	1679 (62.1%)	1994 (66.1%)	0.001
**Primipary**	1212 (44.2%)	1285 (43.3)	0.001
**University degree**	1749 (63.2%)	1979 (65.4%)	0.001
**Salaried work**	2062 (74.5%)	2369 (78.3%)	0.037
**No personal history of any metabolic disease**	2408 (86.5%)	2507 (82.9%)	0.001
**No family history of any metabolic disease**	1259 (45.2)	709 (27.6%)	0.001
**Smoker until /**<br>**during pregnancy**	390 (14%)<br>297 (10.7)	385 (12.8%)<br>292 (9.7%)	 0.001
**Body Weight (Kg) Median (Q_1-Q_3)**<br>**Mean ±SD**	60 (54-68)<br>62.1 ± 11.7	60 (54-67)<br>61.8 ± 11.3	0.452<br>0.362

**BMI (Kg. m^{-2}) Median (Q_1-Q_3)**	22.8 (20.8-25.5)	22.6 (20.6-25.3)	0.092
**Mean ±SD**	23.6 ± 4.2	23.4 ± 4.0	0.071
**TSH (µIU/mL) Median (Q_1-Q_3)**	1.59 (1.05-2.34)	1.78 (1.12-2.59)	0.986
**Mean ±SD**	1.94 ± 1.40	2.05 ± 1.54	0.468
**≥ 2.5 µIU/mL n(%)**	573 (20.7%)	832 (27.5%)	0.001
**T4l (pg/mL) Median (Q_1-Q_3)**	8.67 (7.93-9.48)	8.53 (7.77-9.46)	0.605
**Mean ±SD**	8.70 ± 1.57	8.64 ± 1.49	0.708
**≤7.5 pg/mL n(%)**	404 (14.6%)	581 (19.2%)	0.245
**A-TPO ab (IU/mL) Median (Q_1-Q_3)**	15.9 (1.8-27.2)	8.2 (4.1-25.6)	0.159
**Mean ±SD**	68 ± 137	39 ± 131	0.288
**Use of Iodized salt**	941 (34%)	1238 (40.9%)	0.136

BMI, Body mass index. A-TPO ab, Antithyroid peroxidase antibodies

Table S2. Evolution of gestation by groups (group 2 and 3) comparing women starting levothyroxine treatment (LT) between 10-24 GW with those not receiving LT or starting after GW 24 (no LT), with Group 1 as reference

	GR0UP 1	GROUP 2			GROUP 3		
		LT			LT		
		AFTER GW 9 (n= 279)			AFTER GW 9 (n=50)		
		No LT	Between 10-24 GW	P value	No LT	Between 10-24 GW	P value
**N**	1259	96	183		19	31	
**HPFL**<br>**OR**<br>**95% CI**	419 (33.3)<br>Reference	37 (38.5)<br>1.04<br>0.77-1.40	74 (40.4)<br>1.12<br>0.75-1.49	0.448	10 (52.6)<br>1.93<br>0.54-6.91	15 (48.4)<br>1.55<br>0.64-3.73	0.224
**Miscarriage <12GW**<br>**OR**<br>**95% CI**	20 (1.6)<br>Reference	4 (4.2)<br>1.26<br>0.85-3.07	3 (1.6)<br>1.00<br>0.95-1.08	0.067	2 (10.5)<br>NA	1 (3.2)<br>NA	0.056
**Miscarriage <18GW**<br>**OR**<br>**95% CI**	21 (1.7)<br>Reference	5 (5.4)<br>1.56<br>0.84-5.27	4 (2.2)<br>1.05<br>0.74-1.47	0.512	1 (5.9)<br>NA	2 (6.7)<br>NA	0.321
**Immature <32GW**<br>**OR**<br>**95% CI**	4 (0.3)<br>Reference	3 (3.4)<br>5.08<br>0.71-36.53	3 (1.7)<br>2.78<br>0.92-48.23	0.546	1 (6.3)<br>20.33<br>1.72-239.68	0<br>NA	0.188
**Prematurity <37GW**<br>**OR**<br>**95% CI**	59 (4.9)<br>Reference	11 (13.1)<br>2.40<br>1.31-4.39	15 (8.7)<br>1.21<br>0.88-1.67	0.032	2 (13.3)<br>4.39<br>1.22-15.79	1 (3.6)<br>0.72<br>0.57-1.15	0.031
**VAGINAL N-I**<br>**OR**<br>**95% CI**	813 (60.8)<br>Reference	54 (64.2)<br>1.05<br>0.74-1.47	110 (63.6)<br>1.18<br>0.80-1.75	0.481	11 (68.8)<br>1.69<br>0.59-4.85	20 (71.4)<br>1.11<br>0.80-1.54	0.129
**CS**<br>**OR**<br>**95% CI**	227 (18.0)<br>Reference	22 (22.9)<br>1.10<br>0.73-1.66	35 (19.6)<br>1.07<br>0.75-1.53	0.356	4 (21.1)<br>1.87<br>0.50-7.00	8 (25.8)<br>2.40<br>0.44-13.23	0.267
**GDM**	235 (18.6)	22 (25.2)	32 (18.2)	0.078	4 (25.0)	6 (21.4)	0.369

**OR**<br>**95% CI**	Reference	1.40<br>0.94-2.08	0.74<br>0.47-1.18		1.37<br>0.29-3.50	1.19<br>0.65-2.16	
**Preeclampsia**<br>**OR**<br>**95% CI**	12 (1.0)<br>Reference	2 (2.3)<br>1.42<br>0.15-13.69	3 (1.7)<br>1.38<br>0.62-3.09	0.566	1 (6.3)<br>15.09<br>1.48-153.58	1 (3.6)<br>1.50<br>0.27-8.28	0.304
**SGA/LGA**<br>**OR**<br>**95% CI**	206 (16.9)<br>Reference	21 (24.1)<br>1.06<br>0.75-1.49	44 (25.0)<br>1.27<br>0.90-1.79	0.074	6 (37.5)<br>1.24<br>0.36-4.24	6 (21.4)<br>1.08<br>0.71-1.65	0.580
**Composite AO**<br>**OR**<br>**95% CI**	296 (23.5)<br>Reference	30 (31.3)<br>1.32<br>0.91-1.48	52 (28.4)<br>1.37<br>0.86-2.19	0.058	7 (36.8)<br>1.20<br>0.68-3.77	10 (32.3)<br>1.16<br>0.75-1.81	0.438

Results expressed as n (%). GW, gestation week; LT, levothyroxine treatment; HPFL, History of prior fetal loss**;** CS, Cesarean Section; SGA, Small-for-gestational-age; LGA, large-for-gestational-age; OR (95%CI), Odds Ratio (95% confidence interval) adjusted for age, parity, and smoking habit. Composite AO, Composite Adverse Outcomes: pre-eclampsia, GDM, fetal loss, immature and/or premature newborn, C-section, and newborn of inadequate weight for Gestational age; N.A. no applicable.

Table S3. Women with determination of thyroid hormones prior to 9th GW according to TSH level and initiation of levothyroxine treatment (LT), when indicated, before GW9, and those starting later or not receiving LT.

	TSH < 2.5 µIU/mL	TSH ≥ 2.5 µIU/mL		
		All	Do not start LT before GW9	Start LT before GW9
N	1493	576 (27.8%)	192 (33.3%)	384 (66.6%)
History of prior fetal los n (%)	521 (34.9%)	183 (31.8%)	62 (32.3%)	121 (31.5%)
OR (95%CI)	Reference	0.96 (0.91-1.01)	Reference	0.96 (0.66-1.39)
Fetal loss before 12 GW n (%)	23 (1.5%)	11 (1.9%)	7 (3.6%)	4 (1.0%)
OR (95% CI)	Reference	1.22 (0.58-2.57)	reference	0.54 (0.33-0.92)
Miscarriage before 18 GW n (%)	24 (1.6%)	13 (2.1%)	8 (3.8%)	5 (1.3%)
OR (95% CI)	reference	1.28 (0.58-2.57)	Reference	0.60 (0.35-1.00)
Immature before 32 GW n (%)	9 (0.6%)	4 (0.8%)	4 (2.3%)	0 (0%)
OR (95% CI)	Reference	1.24 (0.38-4.05)	Reference	0.57 (0.63-0.71)
Prematurity before 37 GW n (%)	73 (4.9%)	35 (5.7%)	21 (8.9%)	14 (3.9%)
OR (95% CI)	reference	1.15 (0.75-1.79)	reference	0.78 (0.37-1.64)
CS/ Instrumental n (%)	512 (34.3%)	201 (34.9%)	63 (36.6)	138 (35.9)
OR (95% CI)	reference	1.00 (0.94-1.06)	Reference	0.82 (0.56-1.20)
CS n (%)	272 (18.2%)	107 (18.6%)	38 (19.8%)	69 (17.9%)
OR (95% CI)	Reference	1.02 (0.85-1.23)	Reference	0.91 (0.58-1.43)
Preeclampsia n (%)	13 (0.9%)	10 (1.7%)	7 (3.6%)	3 (0.8%)
OR (95% CI)	Reference	1.43 (0.57-3.61	Reference	0.39 (0.09-1.78)
SGA or LGA n (%)	235 (15.1%)	129 (22.4%)	60 (31.2%)	69 (18.0%)
OR (95% CI)	Reference	1.04 (0.80-1.36)	Reference	0.74 (0.47-1.15)
Composite Adverse Outcomes n(%)	393 (26.3)	165 (28.6)	96 (50.0)	69 (18.0)
OR (95% CI)	Reference	1.07 (0.82-1.39)	Reference	0.71 (0.43-0.91)

LT, levothyroxine treatment; GW, gestational week; CS, Cesarean Section; SGA, Small-for-gestational-age; LGA, large-for-gestational-age; OR (95%CI), Odds Ratio (95% confidence interval) adjusted for age, parity, and smoking habit. Composite Adverse Outcomes: pre-eclampsia, GDM, fetal loss, immature and/or premature newborn, C-section, and newborn of inadequate weight for Gestational age.

Table S4. Women with determination of thyroid hormones prior to 9th GW according to the FT4 level and initiation of levothyroxine treatment (LT) , when indicated, before GW9, and those starting later or not receiving LT.

later or did not receive it.

	FT4 >7.5 pg/mL	T4l ≤ 7.5 pg/mL		
		All	Do not start LT before GW9	Start LT before GW9
N	1648	421 (20.3%)	182 (43.2%)	239 (57.0%)
History of prior fetal los n (%)<br>OR (95% CI)	545 (33.1%)<br>reference	159 (37.8%)<br>1.53 (1.22-1.93)	71 (39.0%)<br>Reference	88 (36.8%)<br>0.98 (0.75-1.28)
Fetal loss before 12 GW n (%)<br>OR (95% CI)	26 (1.6%)<br>Reference	8 (1.9%)<br>1.39 (0.63-3.10)	6 (3.3%)<br>Reference	2 (0.8%)<br>0.57 (0.11-2.87)
Abortion before 18 GW n (%)<br>OR (95% CI)	28 (1.7%)<br>reference	9 (2.1)<br>1.46 (0.68-3.11)	6 (3.3%)<br>Reference	3 (1.3%)<br>0.86 (0.21-3.51)
Immature before 32 GW n (%)<br>OR (95% CI)	7 (0.4%)<br>reference	6 (1.4%)<br>3.90 (1.30-11.68)	4 (2.2%)<br>Reference	2 (0.8%)<br>0.86 (0.16-4.78)
Prematurity before 37 GW n (%)<br>OR (95% CI)	84 (5.1%)<br>reference	24 (5.7%)<br>1.31 (0.82-2.08)	17 (9.3%)<br>Reference	7 (2.9%)<br>0.70 (0.28-1.73)
CS/ Instrumental n (%)<br>OR (95% CI)	561 (34.0%)<br>Reference	152 (36.1%)<br>1.06 (0.87-1.29)	71 (39.0%)<br>Reference	81 (33.9%)<br>0.79 (0.50-1.23)
CS<br>OR (95% CI)	300 (18.2%)<br>Reference	79 (18.8%)<br>1.16 (0.87-1.53)	44 (24.2%)<br>Reference	35 (14.6%)<br>0.80 (0.59-1.00)
Preeclampsia n (%)<br>OR (95% CI)	13 (0.8%)<br>Reference	10 (2.4%)<br>1.48 (0.53-4.13)	9 (4.9%)<br>Reference	1 (0.4%)<br>0.62 (0.57-0.68)
SGA or LGA n (%)<br>OR (95% CI)	287 (17.4%)<br>Reference	77 (18.3%)<br>1.13 (0.85-1.51)	47 (25.8%)<br>Reference	30 (12.6%)<br>0.58 (0.31-0.88)

Composite Adverse Outcomes n(%)	405 (24.6%)	153 (36.3%)	83 (60.1%)	70 (24.9%)
OR (95% CI)	Reference	1.39 (102-1.45)	reference	0.80 (0.66-0.94)

LT, levothyroxine treatment; GW, gestational week; CS, Cesarean Section; SGA, Small-for-gestational-age; LGA, large-for-gestational-age; OR (95%CI), Odds Ratio (95% confidence interval) adjusted for age, parity, and smoking habit. Composite Adverse Outcomes: pre-eclampsia, GDM, fetal loss, immature and/or premature newborn, C-section, and newborn of inadequate weight for Gestational age.
